# What Was First, Obesity or Inflammatory Bowel Disease? What Does the Gut Microbiota Have to Do with It?

**DOI:** 10.3390/nu12103073

**Published:** 2020-10-08

**Authors:** Sara Jarmakiewicz-Czaja, Aneta Sokal, Rafał Filip

**Affiliations:** 1 Medical College of Rzeszow University, Institute of Health Sciences, 35-959 Rzeszow, Poland; asokal@ur.edu.pl; 2 Medical College of Rzeszow University, Institute of Medicine, 35-959 Rzeszow, Poland; r.s.filip@wp.pl; 3 Department of Gastroenterology with IBD Unit, Clinical Hospital No. 2, 35-301 Rzeszow, Poland

**Keywords:** gut microbiota, inflammatory bowel diseases, obesity

## Abstract

A sedentary lifestyle and inadequate nutrition often leads to disturbances in intestinal homeostasis, which may predispose people to excess body weight and metabolic syndrome. Obesity is frequently observed in patients with inflammatory bowel diseases (IBD), similar to the general population. Obesity may exert a negative effect on the course of IBD as well as reduce the response to treatment. Moreover, it may also be an additional risk factor for vein thromboembolism during the flare. In both obesity and IBD, it is of great importance to implement proper dietary ingredients that exert desirable effect on gut microbiota. The key to reducing body mass index (BMI) and alleviating the course of IBD is preserving healthy intestinal microflora.

## 1. Introduction

Year on year, there is an increase in the incidence of inflammatory bowel diseases [1], with most cases reported in North America and Europe. The incidence of inflammatory bowel diseases (IBD) worldwide is growing, and thus driving the health care costs of treating the disease [2]. IBD can occur at any age, but the most frequent morbidity is noted between the twentieth and thirtieth year of life in Crohn’s disease (CD), while in ulcerative colitis (UC) between 30–40 years of age, the second peak of incidence is observed between 60–70 years of age [3]. 

Obesity is characterized by weight gain above normal ranges (body mass index (BMI) ≥30.0 kg/m^2^)), in particular alongside an increase in white adipose tissue (WAT). WAT is divided into subcutaneous, visceral and abdominal, which we divide into subcutaneous, visceral and abdominal [4]. The easiest, fastest and non-invasive way to assess the prevalence of overweight or obesity is the body mass index (BMI) [5].

IBD is often associated with underweight; however, numerous studies indicate an increase in overweight and obesity in CD/UC with the increase in excess body weight. In their work, Harper et al., describe that excessive body weight in IBD increases in parallel [6]. Furthermore, Flores et al., studied a population of people from the United States (US) with diagnosed IBD, and noted obesity in 32.7% of patients, which is analogous to the States US society [7]. In the European cohort, in 2013, obesity occurred in 12% of UC patients and 9% of CD patients [8]. Singh et al., reported that overweight may be present in 20–40% of patients, while obesity may affect even 15–40% [9]. There are also cases of morbid obesity (BMI > 40 kg/m^2^) among patients, where the incidence in IBD is approximately 3.2% [10]. In their study, Ungar et al., noted that 2.3% of patients with CD had a BMI > 35 kg/m^2^, of which 53% were morbidly obese [11]. The case was similar in the study by Causey et al., where 2% of the examined patients had a BMI > 40 kg/m^2^ [12]. Among the therapeutic opportunities in IBD patients with morbid obesity, bariatric surgery is a method of choice; however, its application in CD patients remains questionable [13]. Nevertheless, the results of bariatric surgery in patients with IBD are often comparable to those in healthy subjects [14]. Among patients with CD, the most common is sleeve gastrectomy, while in UC the most prevalent solutions are sleeve gastrectomy and Roux-en-Y gastric bypass [15]. Sharma et al., used the data of IBD patients from 2004–2014 to estimate that despite the increase in morbid obesity in these patients, there was also a decrease in the number of bariatric surgeries. The authors present a relationship with possible further complications (the formation of fistulas or the occurrence of malnutrition) [10].

## 2. Causes of Weight Gain in IBD

The main causes of excess body weight are related to an unsuitable lifestyle, for example, a reduction in the level of physical activity, not only among adults, but also in children and adolescents. On top of that, the consumption of too many calories contributes to the accumulation of excessive amounts of body fat. Particularly common is the consumption of food abundant in sugar, fat or sodium [16]. The causes of obesity may also be existing diseases that cause predisposition to excess body weight. An example of such conditions leading to childhood obesity is Wilms Tumor-Aniridia-Gonadoblastoma-Mental Retardation syndrome (WAGR syndrome) or Prader–Willi syndrome [17]. The existing infrastructure is also increasingly portrayed as one of the factors that has a negative impact on people’s physical activity. Examples here are escalators, elevators and the lack of sidewalks, which makes it necessary to travel by means of transport [18]. Moreover, the degree of education is also a predictor of health, including the occurrence of excess body weight. Cawley shows that both women and men with higher education are less likely to be obese [19]. Barosso et al., draw attention not only to body weight, but above all to changes in body composition in patients after surgical treatment due to refractory IBD. After computed tomography (CT) examination of 22 patients, they observed significant changes in body composition compared to healthy controls. Patients were characterized by increased fat deposition and reduced skeletal muscles. The authors emphasize that patients that are refractory to IBD treatment may have an increased risk of sarcopenic obesity [20,21]. In IBD, sarcopenia may be the result of malnutrition, increased protein breakdown and low protein synthesis, which reduces the amount of myofibrillar proteins. Muscle mass decreases, which can also result in osteoporosis and more frequent bone fractures [22]. The formation and development of sarcopenia in IBD is associated with pro-inflammatory cytokines such as tumor necrosis factor α (TNF-α), interferon-γ, interleukin-Iβ [23]. In their work, Ryan et al., describe that sarcopenia is often observed in the population of IBD patients, which may lead to more frequent surgical interventions in this group. Therefore, the authors recommend screening patients with IBD in this direction [24]. Among drugs routinely used in IBD treatment, systemic steroidotheraphy is one of most common. Obesity is often a side effect of the systemic effects of steroids, which is a risk factor for sarcoidosis [25]. Both as a result of treatment with steroids and biological treatment (e.g., infliximab), there is a change in the distribution of adipose tissue. Parmentier-Decrucq et al., studied 132 patients; however, they noticed response to treatment in 21 people, and eventually included them in the study. Researchers saw an 18% increase in total abdominal mass in the eighth week after introducing the treatment [26]. In addition, the high content of visceral fat in patients with CD is associated with an increased risk of postoperative complications [27,28,29]. Conklin et al., in their systematic review, attempted to define obesity prophylaxis in patients treated with corticosteroids. Most publications described the use of diet and physical activity. However, the authors suggest that high-quality research should be carried out in this area [30].

## 3. Obesity Followed by IBD 

Excessive body weight may be associated with the development of IBD, but research in this area is contradictory. In their review, Harper et al., present that obesity may predispose people to CD, but no such relationship with UC has been demonstrated. In addition, there is an increased risk of other autoimmune diseases in the presence of excess body weight [6]. Similar conclusions were drawn by Khalili et al., who reported that obesity may positively correlate with CD, but not with UC. However, the authors point out that there is need for further research in this direction [31]. Versini et al., indicate one of the reasons to be the secretion of pro-inflammatory cytokines by adipocytes (e.g., interleukin-6 (IL-6), TNF-α). They describe the association of obesity with rheumatoid arthritis (RA), systemic lupus erythematosus (SLE), multiple sclerosis (MS), type I diabetes (T1D-type-1 diabetes) and IBD [32]. However, the direct factor that adversely affects the intestinal microflora, leading to dysbiosis, specifically diet, should also be taken into account. Western-style diets contribute to the reduction and diversity of the commensal microbiota, which may influence the development of IBD. Emerenziani et al., point out that obesity is not only a risk factor for IBD, but also other gastrointestinal diseases, such as gastroesophageal reflux disease, irritable bowel syndrome, diverticulosis, non-alcoholic fatty liver disease and pancreatitis [33]. However, Bilski et al., suggest that, to date, the pathomechanism influencing the occurrence of IBD is unknown, although there are numerous studies linking IBD with excess body weight [34]. On the other hand, Chandrakumar et al., in their work report a much higher incidence of obesity in children and young adults with UC than with CD. Nevertheless, underweight was more frequently observed in newly diagnosed patients with CD [35]. Moreover, the use of total parenteral nutrition in IBD may induce metabolic disorders characteristic of obesity, such as hyperglycemia and hypertriglyceridemia [36,37]. Additionally, this type of nutritional treatment may lead to abnormalities in liver enzymes and to fatty tissue [38].

## 4. Influence of Obesity on the Course of IBD

Obesity increases health care spending as it may predispose patients to type 2 diabetes, heart disease, joint disease, obstructive sleep apnea, and selected neoplastic diseases, including colon cancer [39]. Due to the often comorbid disease entities, together with excess body weight, obese people are at risk of premature death. Hruby et al., show that obesity is one of the strongest predictors of premature mortality [40]. Moreover, the severe course of IBD and the complications of the disease also shorten the life of the patients [41].

### 4.1. Molecular Mechanisms of the Influence of Adipose Tissue on IBD

The presence of a large amount of WAT as a consequence of obesity contributes to stimulating lipolysis and the release of FFA (free fatty acids) by pro-inflammatory cytokines, which is associated with inflammation of WAT. Iyengar et al., suggest that in case of intestinal cancer, inflammation of WAT should be monitored, especially in the case of concomitant obesity [42]. Additionally, in the presence of IBD, co-occurring obesity is not a neutral factor for health. Mesenteric adipose tissue (MAT) plays a special role in the pathogenesis of CD [43]. MAT in CD may be due to adipocyte hyperplasia. Eder et al., show that, as a result of the reduction in the integrity of the mucosal barrier, exposure to microorganisms causes the activation of the transcriptional nuclear factor-kappa B (NF-kB) and the production of pro-inflammatory cytokines. Due to the fact that MAT is composed, in addition to adipocytes, of macrophages, after induction of monocyte chemoattractant protein-1 (MCP-1) expression, tissue infiltration by macrophages occurs. As a consequence, it may determine the severity of the disease and be a significant factor in pathogenesis [44,45,46,47]. More and more researchers draw attention to the link between leptin and IBD. Karmiris et al., indicate that leptin messenger RNA (mRNA) may be overexpressed in the mesenteric WAT, which results in the enhancement of mesenteric TNF-α, thereby enhancing the inflammatory process in the body of IBD patients [48]. Zhao et al., demonstrate the beneficial effects of adiponectin in improving the integrity of intestinal mucosa and alleviating the symptoms of dextran sulfate sodium (DSS) inflammation in Caco-2 cells [49]. A similar conclusion was drawn by Morshedzadeh et al., in their literature review. They established that adiponectin has a protective effect in IBD through, inter alia, inhibition of nuclear factor kappa-light-chain-enhancer of activated B cells (NF-κB) and regulation of interleukin-10 (IL-10) secretion [50]. Obesity may worsen the course of IBD [32]. With concomitant excess body weight, patients with IBD require faster surgical intervention [51]. Furthermore, obesity has a negative impact on the course of IBD itself. Pavelock et al., indicate more frequent hospitalizations of obese patients with IBD compared to those with normal body weight. They also suggest that in individuals with a BMI above 30.0 kg/m^2^, we should consider weight reduction under the supervision of specialists [52]. Similar conclusions were drawn by Yerushalmy-Feler et al., who demonstrated that both low and high BMI upon diagnosis in children was associated with a worse course of illness [53]. Moreover, the study conducted by Yilmaz et al., confirms that BMI and age correlate with the gut microbiota of IBD patients [54].

### 4.2. The Role of Adipose Tissue in the Therapeutic Outcomes of Inflammatory Bowel Disease

Visceral obesity is often closely associated with worse results of surgical treatment in this group of patients. Moreover, the presence of excess body weight may be associated with increased C-reactive protein (CRP) and calprotectin. Additionally, there may be difficulties in carrying out imaging tests for diagnosing or assessing the patient’s health situation [55]. Moreover, obesity has been shown to have an adverse effect on biological therapy. The literature mainly presents such connections for infliximab and adalimumab [56]. Due to increased body weight, there is a faster optimization of the patients’ infliximab levels and therefore a higher dose regimen may be necessary [57]. On top of that, drugs used in IBD such as 5-aminosalicylic acid (5-ASA) derivatives and immunosuppressants (such as azathioprine) may affect the development of pancreatitis and hepatoxicity, which could be more common in the presence of excess body weight [58,59,60,61].

## 5. Intestinal Microbiota, Excess Body Weight and IBD

There are about 10^14^ bacteria and archaea in the human intestinal microbiota [62]. In addition, it has been estimated that the intestinal bacteria contain 100 times more genes than the human genome [63]. Lunch et al., in their work, show, after cataloging over a thousand samples of the human microbiota, the population of the USA, Europe and China, and there are about 9.9 million genes of intestinal microbiome bacteria [64]. 

**Figure 1:**
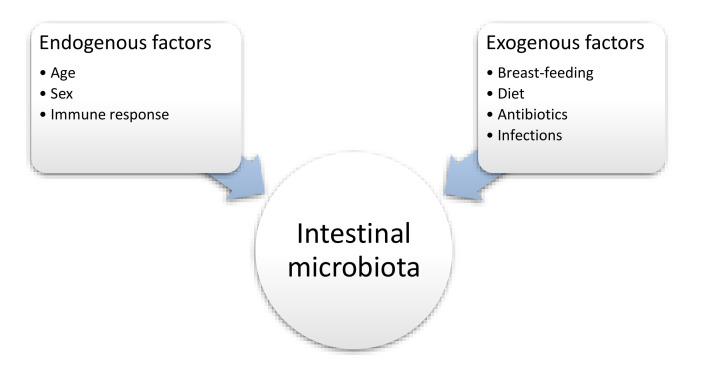
The influence of selected factors on the intestinal microbiota [64].

The human digestive tract is inhabited by microorganisms from the moment of birth and often the child’s intestinal microbiota is similar, in terms of the microbes present, to that of the mother [65]. The amount and quality of microorganisms that exist is individual, but there are several factors that can influence the diversity of the microbiota. The factors include geographic location, genetics and nutritional factors [66,67] (Figure 1).

There are five main phylum of bacteria in the human body: *Bacteroidetes, Firmicutes, Proteobacteria, Verrucomicrobi, Actinobacteria*. There are many links between changes in the microbiota and the incidence of disease [68]. Zhang et al., show that the gut microbiome may influence appetite by modulating leptin, which, in turn, may be indirectly related to excess body weight [69]. Moreover, with obesity, the diversity of the intestinal bacterial population decreases and changes [70]. Qin et al., in their study, describe intestinal dysbiosis in the presence of type 2 diabetes mellitus. They demonstrated, inter alia, a reduction in commensal butyrate-producing bacteria in the intestines (e.g., *Clostridiales, Eubacterium rectale, Roseburia intestinalis*) and an increase in opportunistic microorganisms (e.g., *Bacteroides caccae, Escherichia coli*) [71]. Similarly, obesity showed a lower diversification of the intestinal microflora, especially in butyrate (*Faecalobacterium prausnitzii*) and changes in the proportion of bacteria responsible for the breakdown of mucin in the mucus in the intestines, which makes the body more exposed to pathogenic microorganisms [72]. In the case of coexisting obesity, bacteria of the *Bacteroidetes* and *Firmicutes* phylum change their numbers compared to the intestinal microflora of a person with a normal body weight [73]. Inadequate nutrition has an adverse effect on the intestinal microflora, additionally leading to excess body weight. Chassaing et al., present the effect of selected emulsifiers added to food (carboxymethylcellulose and polysorbate-80) on the intestinal microflora, where they describe the reduction in *Bacteroidetes* and the increase in *Proteobacteria* [74]. Moreover, John et al., pay attention to maintaining the appropriate composition of the diet, which has a beneficial effect on the intestinal microflora. Conversely, a high-fat, sugar-rich diet can increase *Mollicute* and suppress *Bacteroidetes* [75].

Leung et al., in a retrospective study, checked whether obesity in IBD is related to the frequency of *Clostridium difficile* infection. They describe that excess body weight may be associated with a higher incidence of *C. difficile* infection, as is IBD. However, they also emphasize that further research is needed in this area, especially in determining the role of obesity in the process of bacterial acquisition, and thus modulation of disease severity [76]. In IBD, there is a reduction in *Firmicutes* and a quantitative and qualitative change in *Bacteroidetes* [77]. In addition, patients have increased amounts of *Enterobacteriaceae, Proteobacteria*, and *Actinobacteria* [78]. The microbial load is an important factor in influencing the gut microbiota in IBD patients [79]. Due to the reduction in the number of commensal bacteria, the production of short chain fatty acids (SCFA) is also reduced, which are produced from carbohydrates that are not subject to digestive processes in the human body. SCFAs are a medium/nutrient for, inter alia, colonocytes, which show a beneficial effect in maintaining the integrity of the intestinal barrier [80]. Bacteria involved in the production of SCFA (mainly butyrate) are, for example, *Faecalibacterium prausnitzii*, *Eubacterium hallii* and *Eubacterium rectale* [81]. Moreover, some strains of bacteria prefer to metabolize given food components, which, if their amount is increased, may increase the absorption of calories [82]. In addition, bacterial lipopolysaccharide (LPS) may trigger a signal to induce low-grade inflammation that contributes to diabetes, obesity and the metabolic syndrome [83]. In both IBD and obesity, there is an increase in *Proteobacteria, Ruminococcus gnavus* and a decrease in *Clostridium (leptum)* and *Faecalibacterium prausnitzii* [56].

The cause of abnormal intestinal microbiota may be disturbed behavioral rhythm, while intestinal dysbiosis might affect the metabolic pathways and the gut-brain axis, which in turn leads to excessive body weight. However, the authors of the paper indicate that the exact causal factors of this relationship are unclear, and more research should be done in this direction [84]. Similarly, in the case of IBD, Ni et al., demonstrated that it is difficult to explain the exact causal relationship between disease and dysbiosis. The reason for this is the limitations of current research into the gut microbiome. They also indicate that, with the current state of knowledge, more research should be done to fully understand the cause-and-effect relationship that could be used for therapeutic purposes [85]. However, in their work, Bellavia et al., show that it is possible to obtain good therapeutic effects in CD patients with the use of selective antibiotics, which suggests that intestinal dysbiosis may predispose people to the induction of enteritis [86]. 

## 6. The Influence of Selected Nutritional Factors on the Intestinal Microbiota 

**Figure 2:**
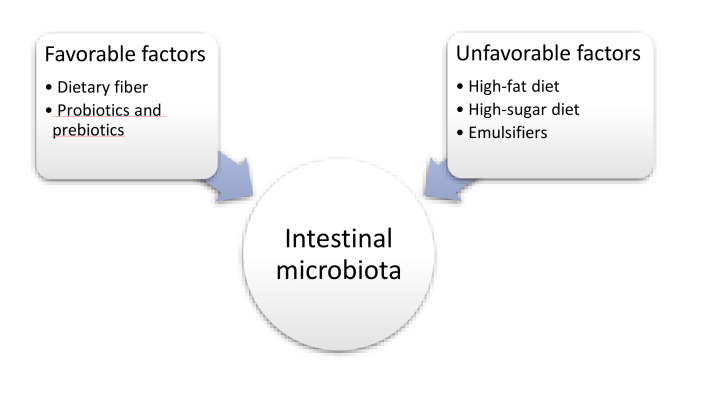
The influence of nutritional factors on the intestinal microbiota.

Individual components of the diet can significantly influence the composition of the intestinal microflora [87] (Figure 2). 

### 6.1. Fat

In numerous studies, the authors present the occurrence of disorders of the intestinal microbiota, including an increase in *Firmicutes, Proteobacteria* and a decrease in *Bacteroidetes* as a result of the use of a high-fat diet [88,89]. A high-fat and protein-rich diet increases the number of bacteria, mainly enterotype 1 and enterotype 3 [90]. Other researchers have shown that such a diet reduces the diversity of the intestinal microflora, e.g., *Actinobacteria* and the species *Eubacterium hallii* and the genus *Megamonas* [91]. Losacco et al., in their study on animal models, presented the effect of a high-fat diet on the activity of transporters of proteins, fats and carbohydrates in the small intestine. They showed that excessive consumption of fat can reduce the expression of protein and carbohydrate transporters (peptide transporter 1 - PEPT1, glucose transporter 2 - GLUT2) and increase sodium transport (sodium–hydrogen exchanger 3 - NHE3). The authors indicated that it may be related to the occurrence of obesity [92]. By contrast, Stenman et al., observed that deoxycholic acid (DCA) has a tissue-degrading effect, adversely affecting the lipid layers and causing intestinal barrier dysfunction. In addition, the authors suggest that prolonged exposure to bile acid may cause mucositis [93]. Fava et al., pay attention not only to the amount of fat in the diet, but also to their quality [94]. Martinez et al., also came to similar conclusions in their review [95]. A menu rich in saturated fatty acids may indirectly activate toll-like receptor 4 (TLR4) and toll-like receptor 2 (TLR2), which may induce inflammation in WAT. In addition, researchers observed that in the case of a diet containing saturated fatty acids, the expression of the C-C motif chemokine ligand 2 (CCL2) chemokine in WAT is higher than in the case of consumption of unsaturated fatty acids [96]. By contrast, Guo et al., in their study, showed that under the influence of a high-fat diet, the amounts of pro-inflammatory cytokines (TNF-α, Interferon gamma - IFN-γ) increase, and the expression of antimicrobial peptides (e.g., regenerating islet-derived 3 gamma - Reg IIIγ, lysozyme) was reduced. The authors primarily suggest that changes in the gut microbiota may cause gut inflammation during coexistence of obesity [97]. On the other hand, inflammation of the intestinal epithelium caused by a high-fat diet may disturb the regulation of food intake and, consequently, lead to the development of obesity. However, Bellavia et al., in their work show that it is possible to obtain good therapeutic effects in CD patients with the use of selective antibiotics, which suggests that intestinal dysbiosis may cause predisposition to the induction of enteritis. A study by Cani et al., showed that the high fat diet (HFD) may affect the development of insulin resistance and inflammation through a mechanism directly related to Gram-negative bacteria, more specifically LPS. Additionally, the supply of the HFD influenced the composition of the microflora by reducing the number of Gram-positive bacteria of the *Bifidobacterium spp.* and *E. rectale - C. coccoides* group. The authors concluded that the gut microflora has a significant influence on the regulation of endotoxemia leading to the development of obesity and/or diabetes [98]. 

### 6.2. Carbohydrates

Non-digestible carbohydrates, i.e., dietary fiber, have a significant effect on maintaining proper intestinal homeostasis. Some bacterial strains are capable of fermenting undigested carbohydrates, such as *Bacteroides, Bifidobacterium* and *Lactobacillus*. They produce substances that can then be used as food for other strains of bacteria [99]. Indirectly, carbohydrates as fermentation products may change the intestinal pH, which predisposes them to change the amount of some bacterial strains (e.g., pH 6.5 was ideal for the development of *Bacteroides*, while at a lower pH it was inhibited) [100,101]. High dietary fiber intake leads to increased production of SCFA, which is the primary source of energy for colonocytes [102]. SCFAs, including butyric acid, may reduce the severity of gastrointestinal pain in patients with UC [103]. In addition, Zhang et al., in their study on animal models, showed that a diet containing dietary fiber in the form of type 2 resistant starch (RS2) can increase the normal function of the intestinal barrier and reduce inflammation in the body. In addition, the authors showed that the supply of RS2 has a beneficial effect on the composition of the intestinal microbiota (the growth of butyrate-producing bacteria: *Ruminococcaceae* decreases *Desulfovibrio* and *Oscillibacter*), thus reducing body weight [104]. Furthermore, dietary fiber can lead to an increase in the production and secretion of intestinal mucus, which is essential for the maintenance of normal intestinal homeostasis [105]. Simpson et al., in their review, presented the effect of dietary fiber on the composition of the intestinal microflora. They noted an increase in the amount of *Prevotella* bacteria and a decrease in *Roseburia* [106]. Moreover, Sinagra et al., in their review, reported that dietary fiber in the diet of patients with CD exerts a beneficial effect on prolonging the remission period in this group of patients [107]. 

### 6.3. Protein

As in the case of fats, individual types and amounts of protein may have a different effect on the composition of the intestinal microflora, its metabolites, and thus on the maintenance of the proper function of the intestinal barrier. Protein that has not been digested is processed by the bacteria into SCFA and hydrogen sulfide (H2S). A large amount of H2S can negatively affect the intestines. Bacteria that can ferment methionine or cysteine are, for example, *Enterobacteria* and *Fusobacteria* [108]. The gut microflora is also directly related to the metabolism of tryptophan. Mills et al., in their work, presented the connection between amino acid metabolism and the functioning of the immune system through ligand-activated aryl hydrocarbon receptor (AhR) signaling. In addition, the authors point to the *Peptostreptococcus russellii* strain and its protective effect on colitis in an animal model due to inducing the production of the metabolite of tryptophan [109]. Another study by Dodd et al., showed the beneficial effects of tryptophan metabolism on the immune system. *C. sporogenes*, via the aromatic amino acid reduction pathway, produces indolepropionic acid (IPA) from tryptophan, which may intensify the intestinal barrier function [110]. The branched chain amino acids (BCAA) found in the diet have properties that enhance the proliferation of intestinal epithelial cells and strengthen the immune system [111]. Lin et al., in their review, point to compelling evidence that there is "collaboration" between certain strains of the gut microflora and the host in metabolizing and using amino acids by the body. The authors also present the relationship between obesity, diabetes and the gut microbiota. Inadequate diet can cause intestinal dysbiosis, and thus lead to a reduction in SCFA synthesis and predisposition to excess body weight [112].

### 6.4. Vitamin D

Vitamin D is one of the factors regulating the body’s immune processes. In a study by Schäffler et al., the effect of oral administration of vitamin D on the intestinal microflora of patients with CD was investigated. They observed that the supplementation of the ingredient resulted in a change in the composition of microflora in patients (the amount of *Bacteroidetes* and *Firmicutes* increased), but the difference was not demonstrated in the control group [113]. Sun, in his work, shows that changes in the intestinal microflora associated with vitamin D may be directly or indirectly involved in a change in the expression of the vitamin D receptor (Vdr) gene [114]. Tabatabaeizadeh et al., indicate that vitamin D deficiency, leading to intestinal dysbiosis, may be a pathogenetic factor in the occurrence of IBD. This may be due to the regulation of angiogenin-4 (an antimicrobial protein) by cholecalciferol [115]. In addition, 1,25-dihydroxyvitamin D (3) deficiencies may reduce the expression of the NOD2 (nucleotide binding oligomerization domain containing 2) gene. It is associated with a reduced activation of nuclear factor kappa-light-chain-enhancer of activated B cells (NF-kB), and thus a reduction in beta-defensin 2—antimicrobial peptide (DEFB2) expression [116]. The authors of the latest systematic review, in their work, show that both the deficiency of the ingredient and its subsequent supplementation have a significant impact on the composition of the intestinal microbiota, but also indicate that further research is necessary due to the limited data on the mechanism of vitamin D’s influence on the gut microbiome [117]. In addition to influencing the composition of the intestinal microflora, vitamin D also improves the function of the intestinal barrier by stimulating the expression of tight junction proteins such as zonula occludens-1 (ZO-1) and claudin-1, which help to maintain normal intestinal homeostasis [118,119]. Naderpoor et al., studied the effect of vitamin D on changing the composition of the intestinal microflora in obese people. Participants in the study received 100,000 international units (IU) of a loading dose (once) followed by 4000 IU / day for 16 weeks and placebo. The researchers observed that *Lachnospira* and *Coprococcus* bacteria grew in the supplement group. The authors found that supplementation with cholecalciferol has a positive effect on the composition of the intestinal microflora in obese people, which may also be helpful in weight reduction [120]. Moreover, Lespessailles et al., in their work, recommend supplementation of 3000 IU of vitamin D in obese patients, both before and after bariatric surgery [121]. By changing the composition of the intestinal microbiota, the decrease in the body’s immunity associated with vitamin D deficiency is also associated with a decrease in the level of vitamin B5, which is produced by bacteria in the gut [122]. Due to the prevalence of vitamin D deficiency in patients with IBD (30%–40%), vitamin D supplementation should be considered [123].

### 6.5. Sweeteners

Suez et al., conducted a study in animal models, investigating the effect of sweeteners on the intestinal microflora. They showed that the consumption of artificial sweeteners can lead to intestinal dysbiosis. In mice, the amount of *Bacteroides* has increased while the amount of *Lactobacillus* has decreased [124]. Another study by Suez et al., pays attention to disturbances in the intestinal microbiota, which may cause symptoms of metabolic syndrome [125]. In a study by Biam et al., acesulfame-K in male mice caused weight gain and an increase in *Bacteroides* four weeks after the start of substance administration, on the other hand, in females, the amount of, among others, *Lactobacillus*. The authors also noted that disturbance of the intestinal microflora by acesulfame-K may be gender-dependent [126]. Xylitol can also alter the composition of the gut microflora. Researchers Uebanso et al., observed that xylitol decreased the amount of *Barnesiella* while it increased *Prevotella* [127]. Daly et al., found that the effect of sweeteners on the composition of the intestinal microflora is selective. They presented this finding on the example of the need for a sweetener membrane receptor (it was saccharin-based dietary sweetener - SUCRAM in the study) for the induction of *Lactobacillus* proliferation [128]. Similar conclusions were obtained by Wang et al., who also indicate that saccharin and acesulfame-K exhibit similar properties of selectively reducing the growth of intestinal bacteria [129]. In addition, selected stevia glycosides (rebaudioside A., stevioside) exhibit *Lactobacillus reuteri* growth inhibitory properties [130]. Magnuson et al., in their literature review, presented that sucralose is not a product that would be metabolized by the intestinal microflora, and thus does not change its composition [131]. However, studies on the effects of sucralose on the composition of the gut microflora are contradictory [132]. The composition of the diet, the dose of sweetener, and the individual predisposition to change the composit. In addition, the use of certain low-calorie sweeteners can lead to impaired glucose tolerance, metabolic syndrome and obesity [133,134].

### 6.6. Emulsifiers, Stabilizers

Chassaing et al., observed that some emulsifiers (polysorbate-80: P80, carboxymethylcellulose: CMC) showed the ability to increase the amount of bacteria adhering to the colon (e.g., mucolytic bacteria *Ruminococcus gnavus*), which could be due to the reduction of the mucus layer. This type of interaction can trigger inflammation. The authors also point to the predisposition of emulsifiers to the occurrence of metabolic disorders [74]. P-80 and CMC can alter bacterial gene expression, which, by increasing flagellin, may lead to inflammation [135]. Glade et al., present in their review that CMC may cause predisposition to bacterial overgrowth in the small intestine, and thus lead to inflammation of the intestine [136]. Jiang et al., have shown, in animal models, that the emulsifier, glycerol monolaurate, can lead to metabolic syndrome, including weight gain. In addition, researchers also observed the formation of intestinal dysbiosis, in which, among others, *Escherichia coli* and *Bacteroides acidifaciens* were found and the *Verrucomicrobia* population decreased [137]. They may also enhance bacterial translocation across the intestinal epithelium, thereby predisposing the patient to enteritis [138]. Low-intensity inflammation can also lead to the development of colorectal cancer [139]. Consuming food rich in emulsifiers can also lead to behavioral changes [140]. Increased feelings of stress in susceptible individuals may be associated with increased consumption of high-calorie foods and thus predispose patients to excess body weight [141].

## 7. Summary

In the light of current research, the answer to the question of what came first—obesity or inflammatory bowel disease—is ambiguous. On the one hand, IBD may have an influence on the development of obesity. Significant differences in the composition of the intestinal microflora are observed in people with excess body weight. An improper diet, rich in saturated fatty acids, sugar or food additives often leads to disturbances in intestinal homeostasis. This, in turn, leads to disturbances in food intake, thus leading to excess body weight.

On the other hand, improper nutrition of people with IBD, chronic pharmacotherapy with glucocorticoids, and limitation of physical activity caused by, for example, surgery, may result in weight gain in patients. Despite the increase in BMI of patients, attention should be paid to abnormalities in the proportion of body weight, i.e., the ratio of adipose tissue to lean body mass, due to the frequent occurrence of sarcopenia, which may correlate with an increase in postoperative complications in patients.
